# A novel TBX5 mutation predisposes to familial cardiac septal defects and atrial fibrillation as well as bicuspid aortic valve

**DOI:** 10.1590/1678-4685-GMB-2020-0142

**Published:** 2020-11-13

**Authors:** Wei-Feng Jiang, Ying-Jia Xu, Cui-Mei Zhao, Xin-Hua Wang, Xing-Biao Qiu, Xu Liu, Shao-Hui Wu, Yi-Qing Yang

**Affiliations:** 1 Shanghai Jiao Tong University, Department of Cardiology, Shanghai Chest Hospital, Shanghai, China.; 2 Fudan University, Department of Cardiology, Shanghai Fifth People’s Hospital, Shanghai, China.; 3 Tongji University School of Medicine, Department of Cardiology, Tongji Hospital, Shanghai, China.; 4 Shanghai Jiao Tong University School of Medicine, Department of Cardiology, Renji Hospital, Shanghai, China.; 5 Fudan University, Cardiovascular Research Laboratory, Shanghai Fifth People’s Hospital, Shanghai, China.; 6 Fudan University, Central Laboratory, Shanghai Fifth People’s Hospital, Shanghai, China.

**Keywords:** Congenital heart disease, atrial fibrillation, bicuspid aortic valve, molecular genetics, TBX5

## Abstract

TBX5 has been linked to Holt-Oram syndrome, with congenital heart defect (CHD) and atrial fibrillation (AF) being two major cardiac phenotypes. However, the prevalence of a *TBX5* variation in patients with CHD and AF remains obscure. In this research, by sequencing analysis of *TBX5* in 178 index patients with both CHD and AF, a novel heterozygous variation, NM_000192.3: c.577G>T; p.(Gly193*), was identified in one index patient with CHD and AF as well as bicuspid aortic valve (BAV), with an allele frequency of approximately 0.28%. Genetic analysis of the proband’s pedigree showed that the variation co-segregated with the diseases. The pathogenic variation was not detected in 292 unrelated healthy subjects. Functional analysis by using a dual-luciferase reporter assay system showed that the Gly193*-mutant TBX5 protein failed to transcriptionally activate its target genes *MYH6* and *NPPA*. Moreover, the mutation nullified the synergistic transactivation between TBX5 and GATA4 as well as NKX2-5. Additionally, whole-exome sequencing analysis showed no other genes contributing to the diseases. This investigation firstly links a pathogenic variant in the *TBX5* gene to familial CHD and AF as well as BAV, suggesting that CHD and AF as well as BAV share a common developmental basis in a subset of patients.

## Introduction

As the most prevalent type of human birth defect, congenital heart defect (CHD) occurs in about 1% of all live neonates, accounting for nearly a third of all forms of developmental abnormalities (Benjamin *et al*., 2019; Oliveira-Brancati *et al*., 2020). Although minor CHD may resolve spontaneously (Benjamin *et al*., 2019), serious CHD may lead to poor health-related quality of life (Amedro *et al*., 2018, 2019; Boukovala *et al*., 2019), reduced exercise capacity (Müller *et al*., 2018; Abassi *et al*., 2019; Smith *et al*., 2019), abnormal nervous develop ment or brain injury (Peyvandi *et al*., 2018, 2019; Khanna *et al*., 2019), hemorrhagic or ischemic stroke (Bokma *et al*., 2018; Giang *et al*., 2018; Pedersen *et al*., 2019), pulmonary hypertension (Brida and Gatzoulis, 2018; Dimopoulos *et al*., 2018; Kaemmerer *et al*., 2018; Pascall and Tulloh, 2018), acute kidney injury or renal dysfunction (Lui *et al*., 2017; Gist *et al*., 2018), infective endocarditis (Jortveit *et al*., 2018; Tutarel *et al*., 2018; Cahill *et al*., 2019), cardiac dysfunction or congestive heart failure (Gilbert *et al*., 2018; Lal *et al*., 2018; Sabanayagam *et al*., 2018; Chan *et al*., 2019), ventricular or supraventricular dysrhythmia (Labombarda *et al*., 2017; Barry *et al*., 2018; Hernández-Madrid *et al*., 2018; Fuchs *et al*., 2019), and death (Lynge *et al*., 2018; Moore *et al*., 2018; Yu C *et al*., 2018). Although vast advance in cardiac surgery allows over 90% of CHD newborns to survive into adulthood, it results in an increasing adult CHD population, and mow CHD adults outnumber CHD children (Bouma and Mulder, 2017; Benjamin *et al*., 2019). Moreover, the late complications and mortality substantially increase in adult CHD patients (Bouma and Mulder, 2017;Spector *et al*., 2018; Trusty *et al*., 2018). Despite clinical importance, the etiologies of CHD in the majority of cases are still elusive.

Cardiogenesis undergoes a highly complex biological process, and both environmental and genetic pathogenic factors can perturb this finely regulated process, leading to CHD (Patel and Burns, 2013; Pierpont *et al*., 2018; Shabana *et al*., 2020). The well-established environmental factors underlying CHD include maternal conditions (such as innutrition, viral infection and endocrine disorder) and exposures to toxic chemicals, therapeutic drugs, or ionizing radiation during pregnancy (Patel and Burns, 2013). However, increasing studies underscore the genetic defects underpinning CHD, and variations in over 70 genes, encompassing those encoding transcription factors, signaling molecules, and sarcomeric proteins, have been involved in CHD (Bashamboo *et al*., 2018; Cantù *et al*., 2018; Jaouadi *et al*., 2018; Li *et al*., 2018a,c; Lombardo *et al*., 2018; Manheimer *et al*., 2018; Pierpont *et al*., 2018; Qiao *et al*., 2018; Razmara and Garshasbi, 2018; Stephen *et al*., 2018; Xu *et al*., 2018; Yu Z *et al*., 2018; Alankarage *et al*., 2019; Gao *et al*., 2019; Kalayinia *et al*., 2019, 2020; Ma *et al*., 2019; Wang J *et al*., 2019, Wang Z *et al*., 2019; Watkins *et al*., 2019; Zhu *et al*., 2019; Faucherre *et al*., 2020; Shabana *et al*., 2020; Zhao *et al*., 2020). Among the recognized CHD-causative genes, the majority code for cardiac transcription factors, encompassing TBX5, GATA4, and NKX2-5 (Li and Yang, 2017). Nevertheless, the genetic determinants underlying CHD in a large proportion of cases remain to be unveiled.

Interestingly, *TBX5* variations have recently been involved in atrial fibrillation (AF), the most common sustained cardiac arrhythmia (January *et al*., 2014). Postma *et al*., (2008) reported that a *TBX5* gain-of-function mutation caused an atypical Holt-Oram syndrome (HOS), with AF being the predominant clinical phenotype. Ma *et al*., (2016) identified multiple loss-of-function mutations in *TBX5* in multiple patients affected with AF. Wang *et al*., (2016) found a novel loss-of-function mutation in *TBX5* in a case with AF. Guo *et al*., (2016) uncovered a new *TBX5* loss-of-function mutation in an index patient with idiopathic AF. These observational results highlight the pronounced genetic heterogeneity of CHD and AF, which makes it justifiable to investigate the prevalence of *TBX5* variations in patients with both CHD and AF, and unveil the molecular mechanism of CHD and AF resulted from novel TBX5 variations.

## Material and Methods

### Study participants

This study subjects comprised 178 unrelated adult patients suffering from both CHD and AF, who were consecutively recruited between February 2015 and March 2019 from the Chinese Han population. Diagnosis of CHD and various kinds of AF was made as described previously (Wang *et al*., 2016; Li *et al*., 2018b; Ma *et al*., 2019). The patients with rheumatic heart disease, ischemic heart disease, essential hypertension, or other recognized risk factors for AF were excluded. The patients with AF occurred after cardiac surgery were also ruled out from the present investigation. If available, the relatives of the probands were also enrolled. The control individuals were 292 unrelated adult healthy persons, who were enlisted from the same geographic area during the same time period. The healthy controls were matched with the affected individuals for ethnicity, sex and age. All study participants were subject to comprehensive medical evaluation, including familial histories, medical histories, physical examination, trans-thoracic echocardiogram, standard 12-lead electrocardiogram, and routine biological tests. This investigation was conducted in accordance with the ethical principles stated in the Declaration of Helsinki. The protocol used in this study was reviewed and approved by the Human Ethics Committee of the Shanghai Chest Hospital, Shanghai, China. Informed consent was obtained from the study participants prior to sample collection.

### Genetic analyses

Blood samples were collected from each study subject. Genomic DNA of each test subject was purified from blood cells with the Wizard Genomic DNA Purification Kit (Promega, Madison, WI, USA). The coding exons and splicing donors/acceptors of *TBX5* were amplified from each study participant’s genomic DNA by polymerase chain reaction (PCR) for a variation scan by PCR-sequencing. The PCR primers were designed as described elsewhere (Zhang *et al*., 2015). Each PCR mixture was prepared in a thin-walled PCR tube with a total volume of 25 μL containing 50 ng of genomic DNA, 0.2 mM dNTPs (Qiagen, Hilden, Germany), 1 × Buffer (Qiagen), 1 × Q solution (Qiagen), 0.5 μM of each primer, and 0.02 U/μL of HotStar Taq DNA Polymerase (Qiagen). PCR was carried out on a Veriti^®^ 96-Well Thermocycler (Applied Biosystems, Foster, CA, USA). The PCR program was set as follows: initial pre-denaturation at 95 °C for 15 min followed by 35 thermal cycles of denaturation at 95 °C for 30 s, annealing at 62 °C for 30 s and extension at 72 °C for 1 min, with final extension at 72 °C for 8 min. The amplified products were fractionated by electrophoresis on a 1.2% agarose gel, and isolated utilizing the QIAquick Gel Extraction Kit (Qiagen). The purified amplicons were subjected to PCR-sequencing under an ABI 3730 XL DNA Analyzer (Applied Biosystems), with the BigDye^®^ Terminator v3.1 Cycle Sequencing Kit (Applied Biosystems) following the manufacturer’s instructions. The detected sequence variant was validated by bi-directional re-sequencing of an independent PCR-generated amplicon from the same subject. For an identified *TBX5* variation, the 1000 Genomes Project database (http://www.1000genomes.org), the Genome Aggregation Database (https://gnomad.broadinstitute.org), and the Single Nucleotide Polymorphism database (http://www.ncbi.nlm.nih.gov/snp) were queried to check its novelty.

In addition, in order to rule out the potential causative effects of other genes on the diseases, whole-exome sequencing (WES) analysis of the mutation carrier’s family members was performed as described previously (Xu *et al*., 2019). In brief, 2 μg of DNA from each family member was utilized to construct an exome library with the SureSelectXT Human All Exon V6 Kit (Agilent Technologies, Santa Clara, CA, USA), and which sequenced on the Solexa Genome Analyzer (GA) IIx platform (Illumina, San Diego, CA, USA), according to the manufacturer’s protocols. Raw image files were processed by the Illumina pipeline to call bases and generate the reads set. By using SOAPaligner, reads were aligned with the human reference genome. Variations of single nucleotide polymorphisms, insertions and deletions were identified by Genome Analysis Toolkit. The identified variants in known genes were classified according to the recommended guidelines (Xu *et al*., 2019). The candidate disease-causing variations found by WES were checked by Sanger sequencing.

### Expression plasmid constructs and site-targeted mutagenesis

The wild-type TBX5 expression plasmid TBX5-pcDNA3.1 was constructed as described elsewhere (Zhang *et al*., 2015). The mutant-type TBX5-pcDNA3.1 was produced via PCR-based site-targeted mutagenesis with a complimentary pair of primers and the QuickChange II XL Site-Directed Mutagenesis Kit (Stratagene, La Jolla, CA, USA) according to the manufacturer’s protocol. The mutant was selected by *Dpn*I (NEB, Hitchin, UK) and was fully sequenced to confirm the desired mutation and to exclude any other unwanted sequence variations. The eukaryotic expression vectors GATA4-pSSRa and NKX2-5-pEFSA, and the natriuretic peptide precursor A-luciferase (NPPA-luc) reporter vector, which expresses Firefly luciferase, were kind gift from Dr. Ichiro Shiojima, at the Department of Cardiovascular Science and Medicine, Chiba University, Japan. The α-myosin heavy chain 6-luciferase (MYH6-luc) reporter plasmid, which expresses Firefly luciferase, was created as described previously (Chen *et al*., 2017).

### Cell culture, plasmid transfection and luciferase analysis

COS-7 cells (derived from the Cell Bank of the Chinese Academy of Sciences, Shanghai, China) were grown in DMEM supplemented with 10% fetal bovine serum (Invitrogen, Carlsbad, CA, USA), as well as penicillin (100 U/mL) and streptomycin (100 μg/mL), in an atmosphere with 5% CO_2_ at 37 °C. COS-7 cells were seeded in 24-well plates, at a density of 2 × 10^5^ per cell before transfection. Plasmids were transfected into cells 24 h after plating with the Lipofectamine 3000 reagent (Invitrogen) according to the product description. To balance transfection efficiency, the internal control plasmid pGL4.75 (Promega), which expresses the Renilla luciferase, was co-transfected. Specifically, COS-7 cells were transiently transfected with empty pcDNA3.1 (1.0 μg), or wild-type TBX5-pcDNA3.1 (1.0 μg), or mutant TBX5-pcDNA3.1 (1.0 μg), or wild-type TBX5-pcDNA3.1 (0.5 μg) plus empty pcDNA3.1 (0.5 μg), or wild-type TBX5-pcDNA3.1 (0.5 μg) plus mutant TBX5-pcDNA3.1 (0.5 μg), together with MYH6-luc (1.5 μg) and pGL4.75 (0.04 μg). To analyze the synergistic transactivation, the same amount (0.6 μg) of each expression vector (empty pcDNA3.1, wild-type TBX5-pcDNA3.1, mutant TBX5-pcDNA3.1, NKX2-5-pEFSA, GATA4-pSSRa) was used singly or in combination, in the presence of NPPA-luc (1.0 μg) and pGL4.75 (0.04 μg). The transfected cells were cultured for 48 h, and then were harvested and lysed. The Firefly luciferase and Renilla luciferase activities were measured under the GloMax-96 Microplate Luminometer (Promega) by utilizing the Dual-Glo Luciferase Assay System (Promega), following the manufacturer’s manual. The activity of the promoter was presented as fold activation (ratio) of Firefly luciferase relative to Renilla luciferase. Each transfection experiment was conducted in triplicate for three times, and the results for promoter activity were given as mean ± standard deviation (SD) of three experiments in triplicate.

### Statistics

Differences in promoter activities between two groups were compared using the Student’s *t*-test, or one-way ANOVA with Tukey’s post hoc test, when indicated, with a *p*<0.05 indicating significant difference.

## Results

### Baseline characteristics of the study patients

In this investigation, a total of 178 unrelated cases suffering from CHD and AF (105 males, with a mean age of 33 years at initial diagnosis of AF) were clinically analyzed in contrast to a total of 292 unrelated control people (173 males, with a mean age of 33 years). The included cases had both echocardiograph-documented CHD and electrocardiogram-documented AF, while the controls had normal echocardiographs and electrocardiograms, with no evidence of cardiac diseases. All the 178 patients had positive family histories of CHD and AF; whereas none of the 292 control individuals had a positive family history of CHD or AF. No study participants had known traditional pathogenic factors for CHD or AF. There was no significant difference between case and control groups in gender, age or ethnicity. The baseline features of the 178 cases affected with CHD and AF are summarized in Table 1.

**Table 1 -:** Demographic and baseline clinical characteristics of the 178 patients with familial congenital heart disease and atrial fibrillation.

*Variable*	*n or mean*	% or range
Demographics		
Male	105	59
Age at initial diagnosis of AF (years)	33 ± 15	14-57
Age at enrollment (years)	45 ± 9	18-65
Distribution of different forms of CHD		
ASD	68	38
VSD	43	24
VSD + ASD	25	14
VSD + PDA	17	10
TOF	11	6
ASD + PDA	10	6
TOF + ASD	4	2
Clinical classification of AF		
Paroxysmal	63	35
Persistent	46	26
Longstanding persistent	39	22
Permanent	30	17
Medical history		
History of cardiac surgery for CHD	51	29
History of catheter ablation for AF	30	17

Data are given as means with standard deviations, number, or percentage. CHD, congenital heart defect; AF, atrial fibrillation; VSD, ventricular septal defect; ASD, atrial septal defect; PDA, patent ductus arteriosus; TOF, tetralogy of Fallot.

###  Detection of a causative *TBX5* mutation 

**Figure 1 -:**
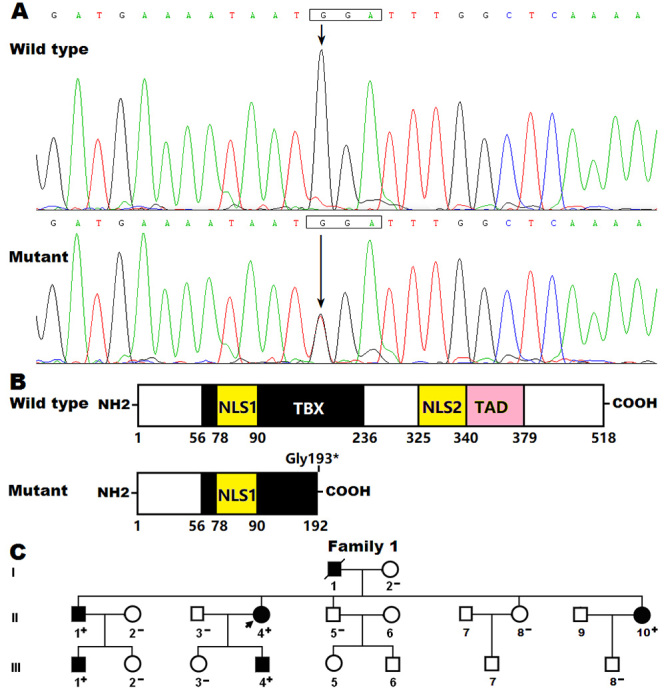
A new *TBX5* mutation responsible for familial heart defect and atrial fibrillation. (A) Sequence chromatograms illustrating the *TBX5* heterozygous mutation from the proband (mutant) and its homozygous wild-type control from a healthy individual (wild type). An arrow points to the heterozygous nucleotides of G/T or the homozygous nucleotides of G/G. (B) Schematic drawings showing the structural domains of the TBX5 proteins. NH2, amino-terminus; NLS1, nuclear location signal 1; TBX, T-box; TAD, transcriptional activation domain; NLS2, nuclear location signal 2; COOH, carboxyl-terminus. (C) Pedigree structure of the family suffering from congenital heart defect and atrial fibrillation. Family members are recognized by generations as well as numbers. Circles mean female members; squares, male family member; closed symbols, affected members; open symbols, unaffected members; the symbol with a slash, the deceased member; the arrow beside the closed square, the index patient; “+”, carriers of the *TBX5* mutation; “-”, non-carriers.

By sequencing the whole coding regions and flanking introns of the *TBX5* gene, a heterozygous variation, NM_000192.3: c.577G>T; p.(Gly193*), was detected in one out of the 178 patients affected with CHD and AF, with an allele frequency of ~0.28% in the patient population. The variation carrier had positive family histories of CHD and AF as well as bicuspid aortic valve (BAV). Genetic studies of the variation carrier’s available family members revealed that the variation co-segregated with ASD and AF as well as BAV, which were transmitted as autosomal dominant traits. In addition, two family members (II-1 and III-1) had also congenital VSD. The sequence chromatograms illustrating the heterozygous *TBX5* variation of c.577G>T and its wild-type control sequence are given in Figure 1A. The schematic diagrams showing the structural domains of wild-type and mutant TBX5 proteins are illustrated in Figure 1B. The pedigree structure of the family with CHD and AF as well as BAV is shown in Figure 1C. The phenotypic characteristics as well as mutational status for TBX5 of the affected family members are presented in Table 2. The nonsense mutation was absent from 296 control people, and was not found in the 1000 Genomes Project database, the Genome Aggregation Database, or the Single Nucleotide Polymorphism database (accessed on May 6, 2020), indicating its novelty. Besides, similar with previous studies (Al-Qattan and Abou Al-Shaar, 2015; Chen *et al*., 2017), no more c.577G>T variation was detected in either cases or controls. Thus, the allele frequency of *TBX5* variation identified in this study was 1/356 (0.28%) in patients and 0/584 (0%) in controls.

**Table 2 -:** Phenotypic features and TBX5 mutation status of the family members with congenital heart defect and atrial fibrillation as well as bicuspid aortic valve.

*Individual*	*Gender*	*Age (years)*	*Cardiac phenotype*	TBX5 mutation p.(Gly193*)
I-1	M	65^*^	ASD, BAV, AF	NA
II-1	M	50	ASD, BAV, AF, VSD	+/-
II-4	F	48	ASD, BAV, AF	+/-
II-10	F	40	ASD, BAV, AF	+/-
III-1	M	24	ASD, BAV, AF, VSD	+/-
III-4	M	22	ASD, BAV, AF	+/-

M, male; F, female; ASD, atrial septal defect; BAV, bicuspid aortic valve; AF, atrial fibrillation; VSD, ventricular septal defect; NA, not available; +/-, heterozygote. ^*^ Age at death.

Additionally, WES analysis of the genomic DNAs from two affected family members (II-4 and III-4) and one unaffected family member (II-3) of the proband who harbored an identified *TBX5* mutation was carried out, and an average of 12,973 exonic variants ranging from 11,652 to 14,395 was detected for each family member. A total of 742 exonic variants were shared by both affected subjects, of which 262 were autosomal, heterozygous non-synonymous, nonsense, and splice site variants. After filtered, only the variation c.577G>T in *TBX5* was verified by Sanger sequencing and demonstrated to co-segregate with CHD and AF as well as BAV in the family.

### No transactivational function of the mutant TBX5 protein

**Figure 2 -:**
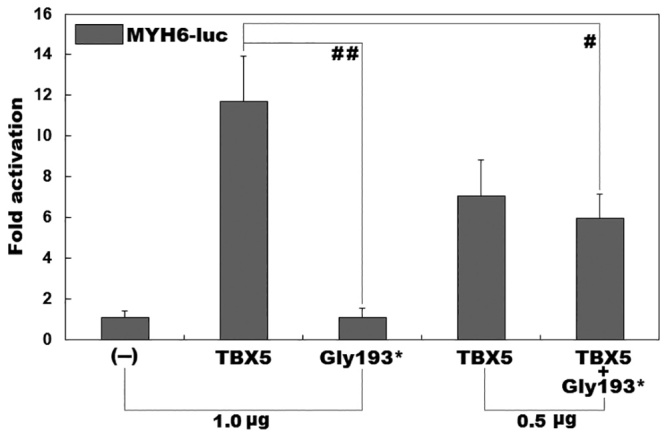
Functional failure of TBX5 caused by the mutation. Activation of α-myosin heavy chain 6 promoter-driven luciferase in cultured COS-7 cells by wild-type or Gly193*-mutant TBX5, singly or together, revealed that the Gly193*-mutant TBX5 protein had no transcriptional activity. Transfection experiments for each plasmid were carried out in triplicates and the results are expressed as means with standard deviations. Here ## and # indicate *p*<0.01 and *p*<0.02, respectively, in comparison with wild-type TBX5.

As shown in Figure 2, the same amount (1.0 μg) of wild-type and Gly193*-mutant TBX5 plasmids transcriptionally activated the *MYH6* promoter by ~12 fold and ~1 fold, respectively (comparison between wild type and mutant: *t* = 8.07389, *p* = 0.00128). When half the amount of wild-type and Gly193*-mutant TBX5 plasmids (each 0.5 μg) was used, the resultant transcriptional activity was ~6-fold (comparison between wild type plus empty plasmid and wild type plus mutant: *t* = 3.91627, *p* = 0.01730).

### No synergistic effect between mutant TBX5 and NKX2-5 as well as GATA4

**Figure 3 -:**
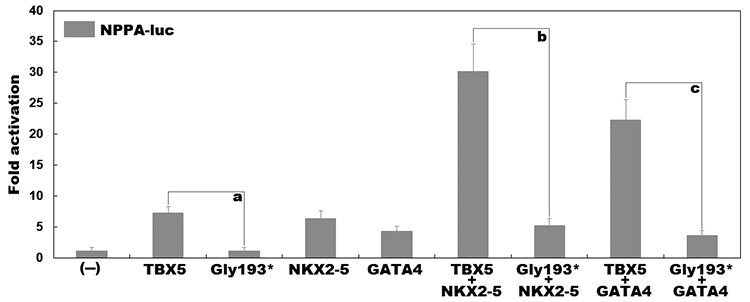
Disrupted synergistic transactivation between mutant TBX5 and NKX2-5 as well as GATA4. The synergistic transactivation of the promoter of natriuretic peptide precursor A in cultured cells by TBX5 and NKX2-5 as well as GATA4 was ablated by the Gly193* mutation. Transfection experiments for each plasmid were done in triplicates, with means and standard deviations shown. Here the symbols a, b and c all indicate *p*<0.001, in comparison with their wild-type counterparts.

As shown in Figure 3, wild-type and Gly193*-mutant TBX5 activated the *NPPA* promoter by ~7 fold and ~1 fold, respectively (comparison between wild type and mutant: *t* = 9.24975, *p* = 0.00076). In combination with wild-type NKX2-5, wild-type and Gly193*-mutant TBX5 activated the *NPPA* promoter by ~30 fold and ~5 fold, respectively (comparison between wild type and mutant: *t* = 9.36360, *p* = 0.00072); while together with wild-type GATA4, wild-type and Gly193*-mutant TBX5 transcriptionally activated the *NPPA* promoter by ~22 fold and ~4 fold, respectively (comparison between wild type and mutant: *t* = 9.51139, *p* = 0.00068).

## Discussion

In the current investigation, a novel heterozygous TBX5 variation, NM_000192.3: c.577G>T; p.(Gly193*), was discovered in a family with CHD and AF as well as BAV. The variation was absent in the 584 reference chromosomes as well as in such population databases as the 1000 Genomes Project database, the Genome Aggregation Database, and the Single Nucleotide Polymorphism database. Functional explorations showed that Gly193*-mutant TBX5 lost transcriptional activity on the *MYH6* and *NPPA* promoters. Moreover, the mutation disrupted the synergistic transcriptional effect between TBX5 and GATA4 as well as NKX2-5. Additionally, WES analysis showed no other genes contributing to the diseases of the family. These observational results indicate that the pathogenic variation in the *TBX5* gene predisposes to CHD and AF as well as BAV.

In humans, *TBX5* is located on chromosome 12q24.1, which encodes a 518-amino acid protein. The TBX5 protein harbors four functionally important domains, including a T-box domain (TBX; amino acids 56-236), which functions to bind target DNAs and interact with other proteins; a transcriptional activation domain (TAD; amino acids 339-379), which is responsible for transactivation of target genes; and two nuclear localization signals (NLS) including NLS1 (amino acids 78-90) and NLS2 (amino acids 325-340), which were essential for nuclear localization (Steimle and Moskowitz1, 2017). Previous studies have corroborated that TBX5 is highly expressed in the hearts of humans and vertebrates, encompassing the endocardium, myocardium, and epicardium of embryonic and adult hearts, and its expression is much higher in atria than in ventricles during embryogenesis, where it plays a key role in cardiovascular morphogenesis and postnatal heart remodeling (Steimle and Moskowitz1, 2017). Recent research has validated that TBX5 transcriptionally regulates expression of many target genes, including *NPPA*, *GJA5*, *MYH6* and *SCN5A*, separately or together with GATA4, GATA6, NKX2-5, MEF2C and TBX20 (Steimle and Moskowitz1, 2017), and variations in TBX5 and its target genes as well as cooperative partners have been reported to result in CHD and/or AF in humans (Postma *et al*., 2008; Mahida, 2014; Guo *et al*., 2016; Ma *et al*., 2016; Wang *et al*., 2016; Li and Yang, 2017; Campbell and Wehrens, 2018). In the current investigation, the pathogenic variation detected in patients with familial CHD and AF as well as BAV was predicted to produce a truncating TBX5 protein with most functional domains lost, and functional explorations revealed that the mutant TBX5 protein failed to transcriptionally activate target genes. Moreover, the pathogenic variation ablated the synergistic transactivation between TBX5 and NKX2-5 as well as GATA4. These data indicate that *TBX5* haploinsufficiency is a molecular mechanism of CHD and AF as well as BAV in a subset of patients.

It might be ascribed to the aberrant cardiovascular genesis that *TBX5* deficiency contributes to CHD and AF. In mice, TBX5 is abundantly expressed in entire cardiac crescent, heart tube, left ventricle, vena cavae, common atrium, and cardiac central conduction system, encompassing atrioventricular bundle and bundle branches (Bruneau *et al*., 1999; Moskowitz *et al*., 2004). Homozygous deletion of *Tbx5* led to murine embryonic death because of failure to undergo cardiac looping as well as left ventricular and sinoatrial hypoplasia; while heterozygous *Tbx5*-bull mice showed ASD, VSD, left ventricular hypoplasia, endocardial cushion defect, and conduction system anomalies, encompassing atrioventricular conduction blocks and bundle branch blocks (Bruneau *et al*., 1999; Bruneau *et al*., 2001; Moskowitz *et al*., 2004). In addition, in murine hearts *Tbx5* haploinsufficiency also markedly reduced the transcription of multiple target genes, including *Nppa* and *Cx40* (Bruneau *et al*., 2001). Moreover, adult-restricted *Tbx5*-mutant mice demonstrated spontaneous AF, and in *Tbx5*-deficient atrial cardiomyocytes, action potential abnormalities occurred due to a decreased SERCA2-mediated sarcoplasmic reticulum calcium uptake (Dai *et al*., 2019). In human beings, TBX5 is highly expressed in embryonic and postnatal hearts (Hatcher *et al*., 2000), and a number of *TBX5* loss- or gain-of-function mutations have been causally linked to HOS, including CHD and AF as well as cardiac block (Al-Qattan and Abou Al-Shaar, 2015). Taken collectively, these findings suggest that genetically defective *TBX5* enhances the susceptibility to CHD and AF in humans, and underscore that TBX5 dosage must be precisely regulated to avoid heart disorders.

Notably, previous studies have causally linked *TBX5* variations to various cardiovascular malformations, including ASD, VSD, atrioventricular septal defect, pulmonary stenosis, hypoplastic left ventricle, mitral valve anomaly (Gharibeh *et al*., 2018). In the current investigation, the affected family members had also BAV, in addition to ASD, VSD and AF, thus expanding the phenotypic spectrum linked to mutant *TBX5*. Given that loss-of-function mutations in multiple transcriptional partners of TBX5 (Balistreri *et al*., 2019), encompassing GATA6 (Gharibeh *et al*., 2018; Xu *et al*., 2018), GATA4 (Yang *et al*., 2017; Li *et al*., 2018c), GATA5 (Padang *et al*., 2012; Bonachea *et al*., 2014; Shi *et al*., 2014), NKX2-5 (Qu *et al*., 2014), and TBX20 (Luyckx *et al*., 2019), have been related to BAV, it is very likely that mutated TBX5 contributes to BAV by reducing expression of the target genes related to BAV morphogenesis in synergy with these partners.

## Conclusions

This investigation causally links TBX5 loss-of-function mutation to CHD, AF and BAV for the first time, which highlights the key role of abnormal cardiovascular development in the pathogenesis of CHD, AF and BAV, implying potential implications for individualized prophylaxis and management of patients with CHD and AF as well as BAV.
